# The link between intrauterine adhesions and impaired reproductive performance: a systematic review of the literature

**DOI:** 10.1186/s12884-022-05164-2

**Published:** 2022-11-14

**Authors:** Angelo B. Hooker, Robert A. de Leeuw, Mark Hans Emanuel, Velja Mijatovic, Hans A. M. Brolmann, Judith A.F. Huirne

**Affiliations:** 1 Department of Obstetrics and Gynecology, Zaans Medical Center, the Netherlands Koningin Julianaplein 58, P.O. Box 210, 1500 EE Zaandam, the Netherlands; 2 Department of Obstetrics and Gynecology, Amsterdam Reproduction & Development Research Institute, Amsterdam UMC, Amsterdam, The Netherlands; 3 Department of Gynecology and Reproductive Medicine, University Medical Center Utrecht, Utrecht, the Netherlands; 4 Department of Reproductive Medicine, Academic Endometriosis Center, Amsterdam University Medical Center, Vrije Universiteit Amsterdam, Amsterdam, The Netherlands; 5 Department of Obstetrics and Gynecology, UMC, Amsterdam Reproduction and Development Research Institute, Vrije Universiteit Amsterdam, Amsterdam, The Netherlands

**Keywords:** Intrauterine adhesions, Asherman syndrome, Reproductive performance, Infertility, Pregnancy complications, Prevention, Adhesiolysis, Pregnancy

## Abstract

**Background:**

Intrauterine adhesions (IUAs) are one of the main reproductive system diseases in women worldwide. Fusion between the injured opposing walls leads to partial-to-complete obliteration of the cavity and/or cervical canal. The main clinical manifestations in case of IUAs are menstrual disturbances, cyclic pain and reproductive disorders. The reproductive outcomes of women with IUAs remain limited and inefficient compared to women without IUAs, even after adhesiolysis. An exact understanding of the underlying mechanisms and processes to explain the compromised reproductive performance and outcomes in case of IUAs are lacking.

**Methods:**

A systematic literature review of MEDLINE-PubMed (1966 to January 2022) and EMBASE (1974 to January 2022) was performed following Preferred Reporting Items for Systematic Reviews and Meta-Analyses (PRISMA) guidelines. Studies were included if they reported underlying causes, related mechanisms and processes to explain the association between IUAs and impaired reproductive performance, pregnancy and obstetric complications.

**Results:**

After an extensive review of the literature, 58 articles were identified reporting underlying mechanisms to explain the association between IUAs and impaired fertility. Intrauterine scarring influences the process of fertilization, reproductive performance and ultimately reproductive outcome. IUAs can disturb the cervico-utero-tubal sperm transport and result in an avascular and unresponsive endometrium with decreased receptivity and thickness. Abnormal decidualization and abnormal trophoblastic infiltration leads to placental attachment disorders. Moreover, the risk for premature delivery, intrauterine fetal growth restriction and fetal anomalies is increased in case of IUAs.

**Conclusion:**

The impact of IUAs on reproductive performance, even after adhesiolysis, is becoming more apparent. The postulated mechanisms to explain the association are related to sperm transport, embryo implantation and placentation. Prevention, by preserving the basal layer of the endometrium is essential. Effective and evidence-based strategies for the prevention of endometrial injury and formation of IUAs, are urgently needed.

**Supplementary Information:**

The online version contains supplementary material available at 10.1186/s12884-022-05164-2.

## Background

Intrauterine adhesions (IUAs) are one of the main reproductive system diseases in women worldwide [1, 2]. IUAs are a consequence of intrauterine injury, which causes destruction to the basal layer of the endometrium [2–6]. Minimal disease is characterized by thin strands of tissue, while severe disease is characterized by complete obliteration of the cavity [7, 8].

**Fig. 1:**
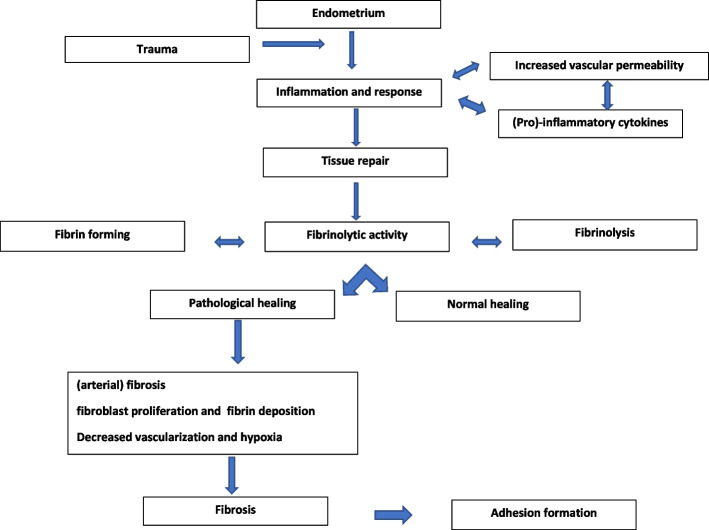
Interconnected processes leading to intrauterine adhesions (IUAs) formation

In 1894, the first case of IUAs was reported [9]. Fifty years later, Asherman [10] described the etiology and symptomatology of IUAs. The definition and terminology used in the literature are inconsistent; initially, the eponymous Asherman syndrome required the identification of pregnancy-related IUAs with signs and symptoms but currently also refers to the presence of IUAs in women without symptoms [2, 11]. The interconnected processes leading to IUA formation are shown in Fig. 1.

IUAs formation is multifactorial with multiple predisposing and causal factors [7, 12]. Pregnancy-related intrauterine surgery is the most important predisposing risk factor and has been reported in up to 91% of IUAs cases [7, 13–16]. IUAs can also develop in the nonpregnant uterus after gynecological procedures [17, 18]. The specific roles of factors such as constitutional characteristics, nutritional status, hypoxia, infection and inflammation have not been completely established yet [4, 11, 19–23].

The main clinical manifestations in cases of IUAs are decreased menstrual or absence of menstrual flow, cyclical abdominal pain and reproductive disorders [4, 24]. It is difficult to establish the exact prevalence of IUAs; it depends on the studied population, diagnostic methods applied, signs and symptoms and degree of awareness [12, 25]. Nevertheless, the prevalence following miscarriage, termination of pregnancy, retained products of conception and myomectomy ranges from 16% to 45.5% [7, 15–21].

There are several mechanisms to facilitate fertilization in the female reproductive tract (Table 1). The impact of intrauterine scarring on fertilization is complex. IUAs can have an adverse effect on fertility, predisposing to pregnancy and obstetric complications in subsequent pregnancies [4, 24, 26, 27]. The aim of the current review is to examine possible mechanisms and (interconnected) processes to explain the compromised reproductive performance and outcomes in the case of pregnancy-related IUAs.

**Table 1:** Mechanism to improve fertilization in the female reproductive tract

Location	Mechanism	Action
**Cervix**	Constituents and architecture of cervical mucus	Increasing penetrability of sperm cells, thereby avoiding immunological defenses
		Sperm cell selection (morphology and motility)
	Mucosal folds in cervical canal/channels	Facilitation of transport of sperm cells through cervical canal toward uterus
**Uterus**	Muscular contractions	Enhance passage of sperm cells, thereby avoiding immunological defenses
		Facilitation of transport of sperm cell through reproductive tract
	Interaction of sperm with uterine cavity	Capacitation and hyperactivation of sperm cells
		Increase in muscular contraction
**Fallopian tubes**	Absence of immunological reaction	Storage of sperm cells
	Detaining sperm cells	Creation of a functional reservoir
		Prolonging survival of sperm cells and improving chances of fertilization
	Activation of sperm cells at time of ovulation	Improving chances of fertilization

## Materials and methods

### Systematic search

We searched MEDLINE (1966 to January 2022) and EMBASE (1974 to January 2022) to identify studies in which fertility, pregnancy and obstetric complications in subsequent pregnancies in cases of pregnancy-related IUAs were reported. The literature review included animal models and human studies.

The following terms were used in the title abstract or as MESH terms: “pregnancy”, “gestation”, “abortion”, “miscarriage”, “ tubal abortion”, “fertility”, “fecundability”, “differential fertility”, “fertility determinant”, “subfecundit*”, “fertility preference”,, “fertility incentive”, “Infertility”, “sterilit*”, “reproductive sterilit*”, “subfertilit*”, “pregnancy Rate”, “pregnancy rate”, “live-Birth Pregnancy Rate”, “reproductive outcome”, “intrauterine adhesion”, “uterine adhesion*”, “asherman syndrome”, “asherman's syndrome”, and “intrauterine synechiae”. The search terms were modified according to the database requirements.

### Paper selection procedure and eligibility

All prospective cohorts, cross-sectional studies, case reports, case series or randomized controlled trials reporting underlying mechanisms and processes between pregnancy-related IUAs and (impaired) fertility, pregnancy disorders and obstetric complications were considered for inclusion. Original articles had to be published as full papers in peer-reviewed journals. Language restrictions were not applied.

Studies were selected independently in a two-stage process. First, eligibility was independently assessed based on the title and abstract by two reviewers (A.H. and R.L). Full manuscripts were obtained for all studies that were selected. In the second step, examination of the full manuscript was carried out to study whether underlying mechanisms and processes in the case of pregnancy-related IUAs were reported. Disagreement was discussed until consensus was reached. The reference lists of the included studies were hand searched for additional relevant studies. Institutional review board approval was not sought since all data were extracted from previously published data. No review protocol exists and the systematic review was not registered.

## Results

**Fig. 2:**
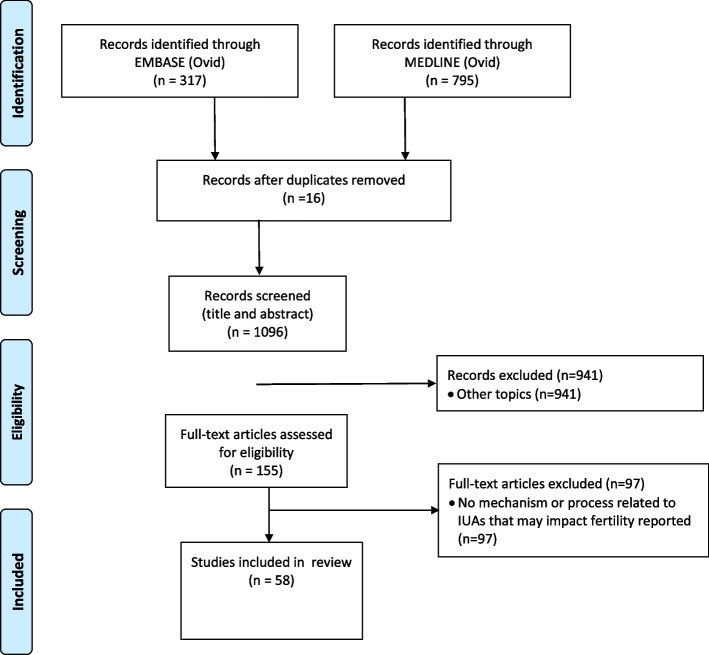
Quorom flow diagram illustrating the selection procedure of relevant articles reporting mechanisms

The search conducted in January 2022 resulted in 795 articles in MEDLINE (Ovid) and 317 articles in EMBASE (Ovid). After removing 16 duplicates, 1096 articles were screened based on their titles and abstracts, and 941 articles were excluded. After screening 155 full texts, 97 papers were excluded. The remaining 58 articles reported underlying mechanisms explaining the association between IUAs and impaired fertility and were included. The flowchart of the study selection is shown in Fig. 2.

### Characteristics of the included studies

The review comprises 24 (systematic) reviews, 17 retrospective and 14 prospective cohort or case control studies, one population based cohort study, three case reports, of which two with review of the available literature and one conference proceeding [see Additional file 1]. The mechanism of impaired reproduction in cases of IUAs are shown in Table 2.

**Table 2:** Mechanisms of impaired reproduction in cases of intrauterine adhesions

Mechanism
**Sperm transport** 1. Occlusion or obstruction of the cervix 2. Alteration of the cervical architecture and canal 3. Decreased quality, composition and amount of cervical mucus 4. Impaired entry of sperm cells 5. Reduced sperm permeability and progression 6. Increased effect of immunological defenses 7. Altered uterine shape 8. Altered contractile activity 9. Deviation and/or obstruction of tubal ostia
**Embryo migration and implantation** 1. Distortion of the uterine cavity 2 Impaired endometrial function, growth and receptivity 3. Alteration to the endometrial and myometrial blood supply 4. Decreased angiogenesis, vascularization and arterial fibrosis 5. Impaired uterine contractions 6. Altered maternal–fetal cross talk
**Placentation** 1. Impaired endometrium function, growth and receptivity 2. Alteration to the endometrial and myometrial blood supply 3. Decreased angiogenesis, vascularization and arterial fibrosis 4. Abnormal decidualization 5. Altered placental implantation and attachment

The reported mechanism in the included articles were related to sperm transport (*n* = 14), embryo implantation (*n* = 30) and placentation (*n* = 17), [see Additional file 1].

### Sperm transport

Sperm transport through the female reproductive tract is essential to achieve a pregnancy. IUAs can disturb the cervico-utero-tubal sperm transport.

The cervix secretes highly hydrated mucus, and sperm cells are quickly guided through the cervix by the microarchitecture of the mucus [28, 29]. Contact of sperm cells with cervical mucus improves penetrability [30]. The folds of the mucosa in the cervical canal form channels during the follicular phase, facilitating sperm transport through the cervix [31]. IUAs can lead to occlusion or obstruction of the endo-cervical opening and/or cervical canal and the architecture may change, resulting in an unresponsive mucosa [32].

The reduction in mucosal folds and channels within the cervical canal reduces entry, hampers sperm transportation and limits the interaction of sperm cells with cervical mucus [33].

A decreased quality, change in composition and amount of cervical mucus limits the permeability and transport of sperm cells into the cervix [28, 30]. When the presence of sperm cells is prolonged in the vagina, the effect of vaginal acidity and immune responses increases and a result, the number of sperm cells available for fertilization decreases [33].

Constriction of the uterus affects the shape and contour of the uterine cavity. In the case of IUAs, the microenvironment within the cavity changes with a negative effect on sperm migration, transport and implantation potential [7, 34–36]. The contractile activity of the uterine muscle enhances the fast passage of sperm cells through the cervix and uterine cavity [33, 35]. Reduced permeability and the prolonged contact of sperm cells within the cavity makes them more prone to immunological defenses [37, 38]. An altered uterine shape has a negative effect on sperm cell migration [35]. Partial-to-complete blockage of the tuba ostia can significantly disrupt and impair sperm cell progression toward the fallopian tubes.

Although tubal abnormalities are a significant cause of infertility, little has been reported on the link between IUAs and tubal abnormalities. One study evaluated 1500 hysterosalpingograms, and among the 92 women whose radiographs showed IUAs, 62 (67.4%) had tubal abnormalities [39]. Once sperm cells have reached the tube, the process of fertilization of the oocyte seems not to be affected.

During transport through the female tract, mature sperm cells become capacitated and hyperactivated, preparing sperm cells to undergo the acrosome reaction and fertilize oocytes [33, 40]. Sperm cells are guided to the oocyte by a combination of thermotaxis and chemotaxis [33]. Whether IUAs could influence the process of capacitation and hyperactivation of sperm cells, which is required for facilitating fertilization, remains unclear as this has not been reported in the literature.

### Embryo implantation

Endometrial receptivity is a process of endometrial maturation during which the blastocyst can attach to the endometrium and may invade the endometrial stroma, a temporal state in which the endometrium is capable of accepting embryo implantation [41, 42]. Implantation is a complex process, a multistep event influenced by hormonal and anatomical adaptation as well as the immune system [43, 44]. IUAs can disturb embryo migration and implantation [45].

**Fig. 3:**
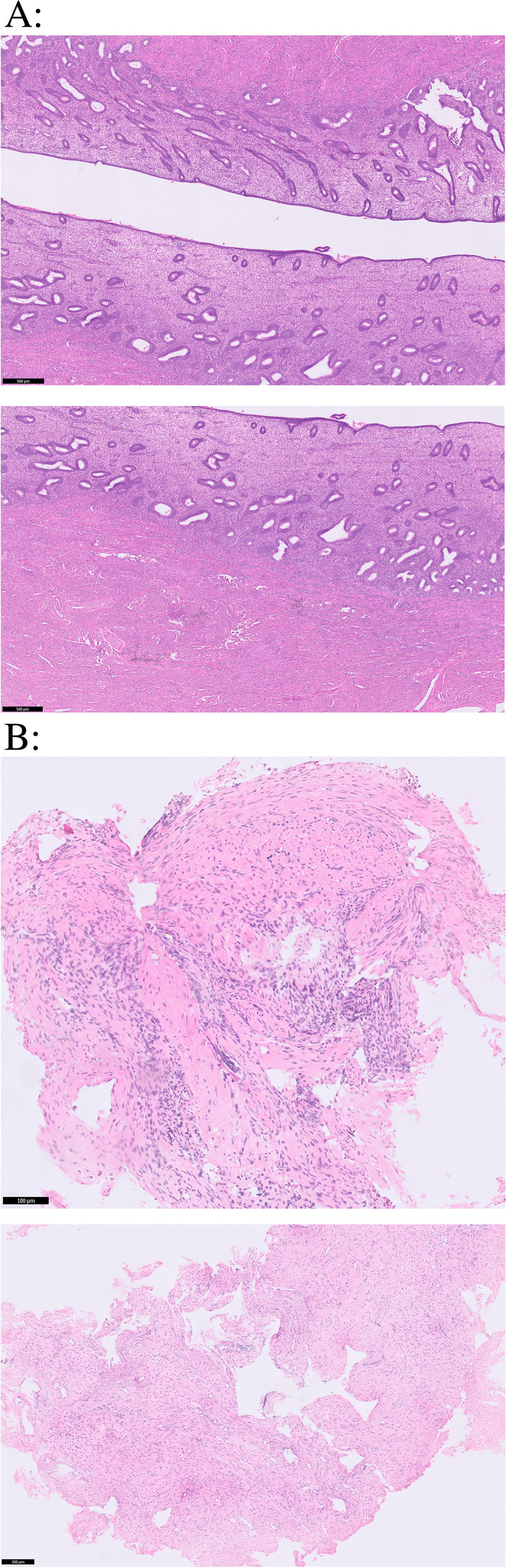
Hematoxylin and eosin (H&E)-stained endometrium. A: Normal endo-myometrial lining, with regularly ranked cubo-columnar endometrial epithelium with a normal functional and basal layer. B: An atrophic endometrium, the endo-myometrial lining is replaced by a band of collagen and fibrotic connective tissue with intrauterine adhesions. of impaired reproductive performance in case of intrauterine adhesions

Cyclic regeneration of the endometrium is regulated and promoted by stem cells (Fig. 3), located near the spiral arteries of the endometrium [46, 47]. The stem cells may differentiate into stromal and epithelial cells and contribute to the maintenance of the endometrium [47, 48].

Damage to the residing stem cells can lead to insufficient replacement of the endometrium and to endometrial insufficiency, resulting in an avascular epithelial monolayer with fibrotic adhesions that are unresponsive to hormonal stimulation [2, 5, 48, 49].

The ability to develop a functional endometrium with correct morphology is crucial for implantation, a damaged endometrium influences endometrial function [45, 50, 51]. A thin endometrium has been identified as an independent and critical factor predisposing the woman to implantation failure [52–54]. Compared to women with a midcycle endometrial thickness > 5 mm, those with a midcycle endometrial thickness ≤ 5 mm have significantly lower pregnancy rates (38% vs. 80%) and significantly higher miscarriage rates (50% vs. 8%) [49]. The endometrium is substantially thinner in women with IUAs than in women without IUAs [51, 55]. Among women with IUAs who underwent IVF, the endometrium was significantly thicker in women who conceived compared to women who did not [56].

In the case of IUAs, the endometrium is characterized by endometrial fibrosis: normal, hormonally responsive endometrial tissue is replaced by atrophic, avascular and unresponsive scar tissue [2, 5, 47, 49, 57]. Both the damaged endometrium and the surrounding endometrium are different from the normal endometrium [23]. There can be a heterogeneous composition wherein inactive and fibrotic endometrium caused by IUAs are adjacent to otherwise healthy endometrium. This leads to a dissociation, there is difference between hormonal stimulation and endometrial response, namely, a secretory or endometrial arrest [57, 58]. This phenomenon is histologically characterized by a combination of simple glands (early secretory phase) and decidualized stroma (late secretory phase) [2, 5, 48, 57].

Fibrosis of the endometrium contributes to impaired reproductive function [2, 59]. The injured and modified endometrium can affect not only implantation but also the preceding period [45]. Endometrial gland secretions contain a variety of proteins essential for survival, growth and development during the early stages of pregnancy, before the establishment of hemotrophic nutrition by the placenta [60]. Angiogenesis and uterine blood flow play a crucial role in supporting endometrial growth [61, 62]. Intrauterine scarring is critical, as it leads to a reduction in blood supply to the surface of the cavity by decreased vascularization [61–63]. Arterial fibrosis contributes to failed endometrial growth by focal ischemia and hypoxia, leading to irreversible changes [64]. Hypoxia seems to cause the uterine cavity to shrink [45, 63].

Throughout the menstrual cycle, endometrial wavelike activity patterns of the uterus have been observed. Subendometrial contraction activity, controlled by steroids, is related to successful reproduction both in naturally and in assisted reproduction cycles [33, 65]. Uterine contractions from the fundus to the cervix are observed primarily in the early to mid-follicular phase, and the contractions draw sperm cells from the cervix into the uterus [35]. The frequency is highest in the peri-ovulatory period, enhancing the rapid passage of sperm through the female tract [66–68]. Seminal components seem to stimulate uterine contractions [33]. In the late follicular phase, the wave pattern is reversed: waves from the cervix to fundus are observed [68–72]. After ovulation, contractions originating in the cervix and fundal area occur simultaneously to prevent the embryo from being expelled from the cervix or tubes and to position the embryo before implantation [67, 69, 73].

Uterine pathology affects peristalsis of the uterus; uncoordinated and impaired uterine contractions seem to play a role in impaired implantation [16, 73]. Sub-endometrial contractions are unfavorably changed or modified in the case of IUAs due to a defect of the endometrial–myometrium interface [74]. Impaired contractions influence and hamper sperm transportation, reduce the permeability of sperm cells toward the fallopian tubes and can lead to impaired transportation of the fertilized oocyte [16, 74]. Impaired placentation due to uncoordinated or altered uterine contractions in association with IUAs is hypothesized but remains uncertain.

Maternal–fetal crosstalk, the complicated process of embryo migration and implantation, is endometrium dependent and affected in the case of IUAs [75–77]. The molecular dialog between the embryo and the receptive endometrium is key for the initiation and progression of implantation [44]. The embryo comes into direct contact with the epithelium, forming initial contacts that are subsequently translated into firm adhesion sites. In the case of IUAs, these processes may be hampered, and defects in the interactions contribute to infertility and implantation failure [75]. Defective vascularization of the (regenerated) endometrium and endometrial arrest could be additional factors [57, 58, 61, 62].

### Placentation

Placental development is a highly regulated process and essential for the development and maintenance of a healthy pregnancy [78]. The placenta plays a central role in the health of both the fetus and mother, fulfills several critical roles as the interface between mother and fetus and has a lifelong impact on their future wellbeing.

Placentation depends on a functional endometrium with correct morphology [49]. In normal placentation, extravillous trophoblasts invade the decidua and convert the spiral arterioles of the endometrium to utero-placental vessels (decidualization); trophoblastic proliferation leads to the formation of chorionic villi [76]. If the underlying endometrium is deficient, decidualization fails, and the trophoblast or chorionic villi invade and penetrate the myometrium, leading to abnormal decidualization and placentation [79, 80].

Abnormal decidualization allows abnormally deep trophoblastic infiltration and abnormal vascularization with secondary localized hypoxia leading to excessive trophoblastic invasion [81, 82]. Villous tissue invades deeply into the myometrium: the myometrial muscle fibers show degenerative changes such as increased fibrous tissue deposits and inflammatory cell infiltration. IUAs lead to scarring of the uterine wall and to disrupted integrity of the endometrial and inner myometrial layers, impairing normal decidualization [7, 79]. Abnormally deep placental anchoring villi and trophoblast infiltration lead to morbid adhesion of the placenta [80]. The extent of endometrial loss increases with IUA severity, which likely confers a greater risk on placental attachment disorders [83].

The mechanism of altered placental implantation is not precisely understood but is a significant source of morbidity and mortality [83, 84]. The absence of the decidua basalis between the chorionic villi and myometrium is a histopathological feature that is pathognomonic of placental attachment disorders but is also characteristic in case of IUAs [85]. The risk of placental implantation disorders is increased in case of IUAs even following adhesiolysis [85, 86]. Whether the lack of decidua is the only factor or others, such as overinvasiveness of trophoblasts and decreased vascularization and angiogenesis of the endometrium and myometrium, are relevant factors remains undetermined.

Altered placentation can lead to obstetric complications such as placental attachment disorders, retained placenta and postpartum hemorrhage [14, 20, 24, 87]. The largest available matched cohort study comparing obstetric outcomes in women with antecedent hysteroscopic adhesiolysis and women without adhesiolysis showed that the risk of placental attachment disorders was significantly increased (OR = 17.93, 95% CI 8.18–39.33), with a significantly higher rate of placenta accreta (OR = 12.69, 95% CI 4.44–33.74) and placenta percreta (OR = 30.74, 95% CI 6.65–142.13) [78]. The rates of placenta previa and retained placenta were also significantly increased (OR = 3.78, 95% CI 1.68–8.47) and (OR = 5.00, 95% CI 3.12–7.89), respectively [79]. Furthermore, the rate of postpartum hemorrhage was significantly increased (OR = 9.33, 95% CI 2.68–32.48), as was the need for blood transfusion (OR = 42.00, 95% CI 5.65–312.2) [79].

There seems to be a link between IUAs and premature delivery, intrauterine fetal growth restriction and fetal anomalies [24, 59, 88, 89]. Defective placentation may lead to intrauterine growth restriction by reducing blood flow to the uterus and placenta. Among women with IUAs, the frequency of low birth weight has been reported to be between 17.9% and 50% [88–90]. However, several small studies have reported no significant differences in birth weight between women with antecedent hysteroscopic adhesiolysis and women without, while others reported a significant association [26, 59, 78]. It remains uncertain whether the reported risk of low birth weight is related to IUAs, abnormal placentation or other (unknown) factors.

## Discussion

IUAs are a cause of significant morbidity [25–27, 91], and the health burden and costs associated with IUAs are substantial [92]. The reproductive outcomes of women with IUAs remain limited and inefficient compared with those without IUAs, even after adhesiolysis [26, 27].

### Findings and interpretation

There is a link between IUAs and impaired reproductive performance [4, 24, 26, 27]. The mechanisms and processes underlying the associations between IUAs and impaired fertility, pregnancy outcomes and obstetric complications have not been reported previously. The presence of IUAs may affect sperm transport, embryo implantation and placentation.

Sperm transport through the female tract can be hampered in case of IUAs, disturbing cervico-utero-tubal sperm transport. IUAs lead to fibrosis and result in an avascular and unresponsive endometrium with diminished function and decreased receptivity and thickness [2, 5, 47–49]. Furthermore, the molecular dialog between the embryo and endometrium is hampered [74–76]. There is an increased risk for abnormal decidualization that allows abnormal trophoblastic infiltration, which leads to placental attachment disorders [44, 79–82]. Moreover, the risk for premature delivery, intrauterine fetal growth restriction and fetal anomalies is increased in cases of IUAs [87–89].

The formation of IUAs seems to be the ultimate result of a process: the culmination of an abnormal response to inflammation, resulting in increased extracellular matrix production with diminished matrix degradation and decreased fibrinolytic activity leading to a defective endometrium and substandard vascularization [93, 94]. Inflammation seems to play a role not only by damaging the endometrium but also by releasing factors that stimulate the formation of fibrotic tissue [13].

### Treatment

Hysteroscopy remains the gold standard to confirm the presence and extent of IUAs; it is an effective method for IUA treatment and follow-up [95]. The aim of adhesiolysis is to restore the functional anatomy of the uterine cavity, to restore endometrial function and increase the chances of becoming pregnant [12, 14, 96]. Nevertheless, adhesiolysis can enhance IUA reformation by destroying the already insufficient endometrium [97]. Prevention of IUA recurrence, reported in up to 66% of patients, remains a clinical challenge [14, 24, 98, 99].

Regeneration and repair of a damaged endometrium remains a clinical challenge. Various cell based therapies have been proposed as an alternative treatment approach in women with IUAs [47, 100, 101]. Stem cells derived from various tissues, platelet-rich plasma and growth factors may be effective alternatives to regenerate the refractory endometrium either through direct endometrium differentiation or paracrine effects [47, 100, 101]. A growing number of studies and clinical trials evaluating the effect and mechanism of cell therapy have been carried out to date; however, there is still no evidence based therapy available [47, 100, 101]. Despite the advances and the promising nature, there are still concerns and challenges that arise when applying cell therapy.

### Prevention

Prevention of IUAs is essential and starts with preserving the basal layer of the endometrium and residing stem cells by reducing trauma [102, 103]. Intrauterine interventions should be prevented as much as possible, and IUA formation should be taken into account when treatment options are discussed. The more intrauterine interventions there are, the more destruction of the endometrium there will be [104]. When there is a need, intrauterine surgery should be performed in the gentlest manner, avoiding unnecessary trauma. The best postoperative management remains unclear, as there is no international protocol or consensus; no single method for preventing recurrence has shown superiority.

Early hysteroscopic intervention seems to prevent adhesion reformation and is associated with a higher cumulative pregnancy and live-birth rate [105, 106]. The adhesive process seems to be progressive and a possible explanation for the high recurrence rate [107]. Unfortunately, the current treatment methods are not optimal, and more effective treatment strategies are needed. Therapeutic options to treat the deficient endometrium and defective vascularization are urgently needed in clinical practice. Future, well-designed and structured studies are necessary to investigate the link between IUAs and compromised reproductive performance to create adequate preventive interventions and treatment strategies to be beneficial in clinical practice.

### Strength and limitations

This is the first published systematic review reporting mechanisms to explain the compromised reproductive performance in case of IUAs. Because of differences and discrepancies in definitions and terminology and lack of knowledge, important causes and mechanisms might not have been reported. Unfortunately, assessment of the risk of potential bias for the individual studies could not be assessed. Nevertheless, understanding of the underlying mechanisms are important from a clinical perspective. A uniform and evidence-based classification system for IUAs is required and essential to enable the evaluation, outline prognosis and efficacy of treatment modalities.

Whether IUAs affect quality of life, sexual functioning and physical and mental health could not be determined. No studies have examined the effects of IUAs on quality of life, sexual functioning and physical and mental health, although in theory, this may play an important role in impaired fertility. IUAs may lead to abnormal uterine bleeding and pelvic pain and therefore might have a negative impact on wellbeing and on physical and mental health [4, 24]. This may lead to avoidance of sexual relations, a decrease in genital sexual behavior and reduced arousal. Whether IUAs affect sexual functioning remains undetermined.

## Conclusions

IUA development is a significant, poorly understood cause of significant morbidity linked to the disruption of the basal layer of the endometrium, a condition with a high impact on female reproduction, adversely affecting reproductive outcomes. Pregnancy may be complicated by placental morbidity, increasing obstetric complications. The impact of intrauterine scarring on fertility is complex, and understanding the related causes and mechanisms of impaired reproductive performance is extremely important.

The impact of IUAs on reproductive performance, even after adhesiolysis, is becoming more apparent. A central but yet unresolved question is how to maintain cavity integrity in the management of IUAs. Evidence-based strategies for prevention are urgently needed. Regeneration of the injured endometrium could be a specific therapy but needs further exploration.

## Supplementary Information

**Additional file 1: Additional table 1**. Included studies in this review.

## Data Availability

All data and materials are published. No new materials were generated for the Review.

## Abbreviations

CI: Confidence interval
IUAs: Intrauterine adhesions
IVF: In vitro fertilization
OR: Odds ratio
vs: Versus
