# Supplementary material for: The link between intrauterine adhesions and impaired reproductive performance: a systematic review of the literature
Source: BMC Pregnancy Childbirth. 2022 Nov 14;22:837. doi: 10.1186/s12884-022-05164-2 (PMC9664654; doi:10.1186/s12884-022-05164-2)
Supplement: Supplementary file 1 — Additional file 1: Additional table 1. Included studies in this review. [file 12884_2022_5164_MOESM1_ESM.docx]

**Additional table 1. Included studies in this review.**

| **First author, year of publication** | **Title** | **Study design** | **Focus of study** | **Mechanism** |
| --- | --- | --- | --- | --- |
| Deans & Abbott. (2010) [2] | Review of intrauterine adhesions. | Review of the literature | Review of the literature on symptomatic and asymptomatic intrauterine adhesions. | S, E |
| Salazar *et al.* (2017) [5] | A comprehensive review of Asherman's syndrome: causes, symptoms and treatment options. | Review of the literature | Understanding the cause of intrauterine adhesions and the common clinical presentation. | E |
| Schenker & Margalioth (1996) [7] | Intrauterine adhesions: an updated appraisal. | Review of the literature | A review on the incidence, etiology, symptomatology, pathology, and treatment of intrauterine adhesions | P |
| Valle & Sciarra. (1988) [14] | Intrauterine adhesions: hysteroscopic diagnosis, classification, treatment, and reproductive outcome. | Retrospective case series | To assess therapeutic prognosis according to the extent of uterine cavity occlusion by hysterosalpingography and the type of intrauterine adhesions observed at hysteroscopy. | P |
| March. (2011) [16] | Asherman's syndrome. | Review of the literature | Review of the literature on intrauterine adhesions. | E |
| Chen *et al.* (2017) [20] | Prevalence and Impact of chronic endometritis in patients with intrauterine adhesions: a prospective cohort study. | Prospective cohort study | To evaluate the prevalence and impact of chronic endometritis in women with intrauterine adhesions. | P |
| Evans-Hoeker & Young (2014) [23] | Endometrial receptivity and intrauterine adhesive disease. | Review of the literature | Review of the literature on endometrial receptivity and intrauterine adhesions. | E |
| Capella-Allouc (1999) [24] | Hysteroscopic treatment of severe Asherman's syndrome and subsequent fertility | Retrospective case study | Evaluation of the efficacy of hysteroscopic adhesiolysis in patients with severe Asherman's syndrome. | P |
| Hooker *et al.* (2021) [26] | Reproductive performance of women with and without intrauterine adhesions following recurrent dilatation and curettage for miscarriage: long-term follow-up of a randomized controlled trial. | Prospective case control study | To compare reproductive performance of women with identified and treated IUAs versus women without IUAs. | P |
| Katz *et al.* (1997) [28] | Analysis of preovulatory changes in cervical mucus hydration and sperm penetrability. | Prospective cohort study | To analyze changes in cervical mucus occurring during the proliferative phase of the menstrual cycle. | S |
| Katz *et al.* (1978) [29] | The movement of human spermatozoa in cervical mucus. | Prospective case control study | Movement characteristics of freely swimming spermatozoa were studied. | S |
| Morales *et al.* (1993) [30] | Human cervical mucus: relationship between biochemical characteristics and ability to allow migration of spermatozoa. | Prospective case control study | To study the capacity of mucus to allow sperm migration. | S |
| Mullins and Saacke. (1989) [31] | Study of the functional anatomy of bovine cervical mucosa with special reference to mucus secretion and sperm transport. | Prospective cohort study | The bovine cervical mucosa was investigated with respect to structure, mucus secretory pattern, and sperm transport. | S |
| Magos*.* (2002) [32] | Hysteroscopic treatment of Asherman's syndrome. | Review of the literature | Review of the literature on intrauterine adhesions. | S |
| Suarez & Pacey*.* (2006) [33] | Sperm transport in the female reproductive tract. | Review of the literature | Passage of sperm through the female reproductive tract | S, E |
| Harada *et al.* (2016) [34] | The impact of adenomyosis on women's fertility. | Review of the literature | The impact of adenomyosis on women's fertility. | S |
| Fukuda & Fukuda*.* (1994) [35] | Physiology: uterine endometrial cavity movement and cervial mucus. | Prospective comparative study | To observe uterine endometrial movements during mid- and late follicular and luteal phase | S |
| Tan & Robertson. (2011) [36] | The role of imaging in the investigation of Asherman's syndrome. | Case report Review of the literature | A case is presented and search of the literature on diagnostic imaging of Asherman's syndrome and intrauterine adhesions | S |
| Suares & Oliphant. (1982) [37] | Interaction of rabbit spermatozoa and serum complement components. | Prospective cohort study | To determine complement activation by rabbit spermatozoa. | S |
| Dostal *et al.* (1997) [38] | Inhibition of bacterial and boar epididymal sperm immunogenicity by boar seminal immunosuppressive component in mice. | Prospective cohort study | To determine the effect of the immunosuppressive component of boar seminal vesicle secretion | S |
| Olawale *et al.* (2014) [39] | Tubal abnormalities in patients with intrauterine adhesion: evaluation using hysterosalpingography. | Retrospective case serie | To determine the prevalence of tubal abnormalities among patients with intrauterine adhesion. | S |
| De Jonge. (2005) [40] | Biological basis for human capacitation. | Review of the literature | To present a framework for the changes human spermatozoa may experience as they make their way through the reproductive tract. | S |
| Paria *et al.* (2001) [44] | Implantation: molecular basis of embryo-uterine dialogue. | Review of the literature | A review article on implantation, dedicated to Ann McLaren for her contribution in the field of preimplantation and implantation biology | E |
| Malhotra *et al.* (2012) [45] | Changes in endometrial receptivity in women with Asherman’s syndrome undergoing hysteroscopic adhesiolysis. | Prospective cohort study | To determine whether there is any improvement in the endometrial receptivity in infertile women with Asherman's syndrome undergoing hysteroscopic adhesiolysis. | E |
| Lv *et al.* (2021) [47] | Adult stem cells in endometrial regeneration: molecular insights and clinical applications. | Review of the literature | A review of the biological mechanisms of endometrial repair and research in adult stem cell therapy for damaged endometrium | E |
| Gargett *et al.* (2016) [48] | Endometrial stem/progenitor cells: the first 10 years. | Review of the literature | To summarize the identification and characterization of endometrial stem/progenitor cells | E |
| Baradwan *et al.* (2018) [49] | The effect of endometrial thickness on pregnancy outcome in patients with Asherman's syndrome post-hysteroscopic adhesiolysis. | Retrospective case control serie | To determine whether endometrial thickness could influence pregnancy outcome of hysteroscopic adhesiolysis in women with a history of Asherman's syndrome. | E |
| Pabuçcu *et al.* (1978) [50] | Hysteroscopic treatment of intrauterine adhesions is safe and effective in the restoration of normal menstruation and fertility. | Retrospective  case serie | To assess the safety and efficacy of hysteroscopic adhesiolysis in patients with recurrent pregnancy loss and infertility. | E |
| Lo *et al.* (1978) [51] | Endometrial thickness measured by ultrasound scan in women with uterine outlet obstruction due to intrauterine or upper cervical adhesions. | Prospective cohort study | To test the hypothesis that women with localized adhesions occluding only the uterine outlet will have much thinner endometrium than controls. | E |
| Gleicher *et al.* (2011) [52] | Successful treatment of unresponsive thin endometrium. | Prospective cohort study | To assess whether inadequate, thin endometrium (<7 mm), after failure to standard treatment will be respond to cytokine treatment. | E |
| Zhao *et al.* (2012) [53] | The effect of endometrial thickness and pattern measured by ultrasonography on pregnancy outcomes during IVF-ET cycles. | Prospective cohort study | To study the effect of endometrial thickness and pattern upon pregnancy outcomes in patients undergoing IVF-ET. | E |
| Fang *et al.* (2016) [54] | The effect of endometrial thickness on the day of hCG administration on pregnancy outcome in the first fresh IVF/ICSI cycle | Retrospective case serie | To investigate the effect of endometrial thickness on human chorionic gonadotropin day on in IVF/ICSI outcome. | E |
| Myers & Hurst. (2012) [55] | Comprehensive management of severe Asherman syndrome and amenorrhea. | Retrospective case serie | To describe a comprehensive approach and pregnancy outcome in women with severe Asherman syndrome and amenorrhea. | E |
| Wang *et al.* (2019) [56] | Factors affecting pregnancy outcomes following the surgical removal of intrauterine adhesions and subsequent in vitro fertilization and embryo transfer. | Retrospective case series | To investigate clinical factors affecting pregnancy rates following surgical removal of intrauterine adhesions and IVF-ET. | E |
| Emanuel. (2015) [57] | Endometrial secretory arrest in patients with asherman syndrome: a new challenge or chance? | Conference proceeding | To investigate the endometrium of Asherman Syndrome representative endometrial biopsies were examined. | E |
| Movilla *et al.* (2020) [58] | Endometrial thickness measurements among Asherman syndrome patients prior to embryo transfer. | Retrospective case serie | To examine whether there is an association between endometrial thickness and clinical pregnancy rate among Asherman syndrome patients utilizing IVF and embryo transfer | E |
| Baradwan *et al.* (2018) [59] | The birth weight in pregnant women with Asherman syndrome compared to normal intrauterine cavity: a case-control study. | Retrospective case control study | To determine the association between low birth weight in women with Asherman syndrome after hysteroscopic adhesiolysis and women who had normal intrauterine cavity post hysteroscopy. | E, P |
| Smith. (1998) [61] | Angiogenesis, vascular endothelial growth factor and the endometrium. | Review of the literature | A review of the literature on angiogenesis and the expression of endothelial growth factor in the human endometrium. | E |
| Smith (2000) [62] | Angiogenesis and implantation. | Review of the literature | The development of blood vessels in the pre-implantation phase and the complex interplay that leads to the establishment of a healthy placenta are reviewed. | E |
| Zhao *et al.* (2020) [63] | Cold scissors ploughing technique in hysteroscopic adhesiolysis: a comparative study. | Retrospective case control study | The study aimed to assess the efficacy, feasibility, and safety of hysteroscopic adhesiolysis using cold scissors technique. | E |
| Fletcher *et al.* (2008) [64] | Hypoxia-generated superoxide induces the development of the adhesion phenotype. | Prospective cohort study | To determine the mechanisms by which hypoxia induces the development of adhesion phenotype in normal peritoneal fibroblasts. | E |
| Bulletti & Ziegler. (2006) [65] | Uterine contractility and embryo implantation. | Review of the literature | To assess the importance of uterine contractility in the implantation of human embryos. | E |
| Kuijsters *et al.* (2017) [73] | Uterine peristalsis and fertility: current knowledge and future perspectives: a review and meta-analysis. | Review of the literature | To provide an overview of the current knowledge on uterine peristalsis. | E |
| Shokeir *et al.* (2008) [74] | The nature of intrauterine adhesions following reproductive hysteroscopic surgery as determined by early and late follow-up hysteroscopy: clinical implications. | Retrospective case serie | To evaluate the rate and characteristics of postoperative intrauterine adhesions that might be formed following hysteroscopic reproductive surgery. | E |
| Revel. (2012) [75] | Defective endometrial receptivity. | Review of the literature | To review factors which might cause defective endometrial receptivity. | E |
| Gellersen & Brosens. (2014) [76] | Cyclic decidualization of the human endometrium in reproductive health and failure. | Review of the literature | To summarize the mechanisms that govern differentiation of endometrial stromal cells and the role of decidualization. | E |
| Evans *et al.* (2016) [77] | Fertile ground: human endometrial programming and lessons in health and disease. | Review of the literature | To present the current knowledge regarding the normal and abnormal function of the human endometrium. | E |
| Feng *et al.* (2020) [78] | Obstetrical outcome in the third trimester after hysteroscopic adhesiolysis. | Retrospective  case serie | To investigate the obstetrical outcome in the third trimester of women who previously underwent hysteroscopic adhesiolysis. | P |
| Zhang *et al.* (2020) [79] | The incidence of placenta related disease after the hysteroscopic adhesiolysis in patients with intrauterine adhesions. | Retrospective case serie | To analyze the correlation between placenta related disease of pregnant women with antecedent hysteroscopic adhesiolysis due to intrauterine adhesions. | P |
| Eller *et al.* (2009) [80] | Optimal management strategies for placenta accreta. | Retrospective cohort study | To determine which interventions for managing placenta accreta were associated with reduced maternal morbidity. | P |
| Jauniaux & Jurkovic. (2012) [81] | Placenta accreta: pathogenesis of a 20th century iatrogenic uterine disease. | Review of the literature | To review the current literature on the etiology, pathophysiology and early prenatal diagnosis of placental accreta. | P |
| Sonan *et al.* (2018) [82] | Placenta accreta following hysteroscopic lysis of adhesions caused by Asherman's syndrome: a case report and literature review. | Case report Review of the literature | A case of placenta accreta following hysteroscopic lysis of adhesions caused by Asherman's syndrome and IVF treatment is reported and the literature on placenta accreta following hysteroscopic adhesiolysis is reviewed. | P |
| Kim *et al.* (2019) [83] | The management and outcomes of placental adhesion. | Review of the literature | To review the available literature on the management of placental adhesion. | P |
| Khopkar *et al.* (2006) [84] | Morbid adhesion of the placenta after hysteroscopic lysis of intrauterine adhesions. | Case report | To report a case of morbidly adherent placenta in a patient who had previously undergone hysteroscopic lysis of intrauterine adhesions. | P |
| Upson *et al.* (2014) [85] | Placenta accreta and maternal morbidity in the Republic of Ireland, 2005–2010. | Population  based cohort study | To describe the nationwide prevalence of placenta accreta and to quantify its impact on maternal morbidity. | P |
| Jauniaux *et al.* (2016) [86] | Accreta placentation: a systematic review of prenatal ultrasound imaging and grading of villous invasiveness. | Review of the literature | To evaluate the various ultrasound signs proposed in the international literature for the prenatal diagnosis of accreta placentation and assessment of the depth of villous invasiveness. | P |
| Zikopoulos *et al.* (2004) [28] | Live delivery rates in subfertile women with Asherman's syndrome after hysteroscopic adhesiolysis using the resectoscope or the Versapoint system. | Retrospective cohort study | To report on a 10-year experience in the treatment of subfertile women with intrauterine adhesions using the resectoscope or the Versapoint system. | P |
| Yu *et al.* (2008) [88] | Factors affecting reproductive outcome of hysteroscopic adhesiolysis for Asherman's syndrome. | Retrospective cohort study | To evaluate the outcome of hysteroscopic adhesiolysis in women with Asherman's syndrome. | P |

S: sperm transport; E: embryo implantation; P: placentation.
